# Effects of 17β-estradiol on proliferation, cell viability and intracellular redox status in native human lens epithelial cells

**Published:** 2011-07-20

**Authors:** D. Celojevic, A. Petersen, J-O. Karlsson, A. Behndig, M. Zetterberg

**Affiliations:** 1 Institute of Neuroscience and Physiology, Department of Clinical Neuroscience and Rehabilitation/Ophthalmology, The Sahlgrenska Academy at University of Gothenburg, Gothenburg, Sweden; 2 Institute of Biomedicine, Department of Medical Chemistry and Cell Biology, The Sahlgrenska Academy at University of Gothenburg, Gothenburg, Sweden; 3 Department of Clinical Sciences/Ophthalmology, Umeå University, Umeå, Sweden

## Abstract

**Purpose:**

The purpose of this study was to examine the effects of 17β-estradiol on proliferation, cell death and redox status in cultured human lens epithelial cells (HLECs).

**Methods:**

HLECs were exposed to 17β-estradiol after which cell viability was measured by 3-(4,5-dimethylthiazolyl-2)-2,5-diphenyltetrazolium bromide (MTT) and the number of mitotic and apoptotic cell nuclei was determined after staining with Hoechst 33342. Apoptosis was also determined by measuring caspase-3 activity and propidium iodide was used to determine the proportion of non-viable cells. Pro- and antioxidative effects of 17β-estradiol was investigated by measuring peroxides, superoxides and glutathione, using dichlorofluorescein diacetate (DCFH-DA), dihydroethidium (HET), and monochlorobimane (MCB), respectively. Effects on mitochondrial membrane potential were determined using 5,5′,6,6’-tetrachloro-1,1’,3,3′- tetraethylbenzimidazolylcarbocyanine iodide (JC-1). The ability of 17β-estradiol to prevent reactive oxygen species (ROS)-production in HLECs after exposure to 25 µM H_2_O_2_ for 24h was also measured.

**Results:**

This study demonstrates increased mitotic activity in HLECs exposed to physiologic concentrations of 17β-estradiol (1 nM). Pharmacological concentrations of 17β-estradiol caused increased number of apoptotic cell nuclei and caspase-3 activation. Physiologic concentrations of 17β-estradiol (0.1–10 nM) stabilized the mitochondrial membrane potential. Similar or slightly higher concentrations of 17β-estradiol (0.01–1 µM) protected against H_2_O_2_-induced oxidative stress as evident by decreased levels of peroxides and superoxides.

**Conclusions:**

The present study demonstrates mitogenic and anti-oxidative effects of 17β-estradiol at physiologic concentrations, whereas pharmacological levels induced oxidative stress and acted pro-apoptotic in cultured lens cells.

## Introduction

Several studies indicate a higher prevalence of cataract among women as compared to men at the same age. Epidemiologic studies and data from National Quality Registers demonstrate a higher incidence of cataract extraction in women [1,2]. It has been suggested that there are gender-related differences in self-assessment of visual function and/or different demands for good visual acuity for men and women depending on their respective everyday activities or differences in longevity, which could contribute to this difference [2,3]. However, several population-based studies report on higher prevalence of lens opacities in women [4-7], thus indicating that female gender is indeed a “true” risk factor for cataract.

There is accumulating evidence that hormonal status and the duration of life-time exposure to estrogen influence the risk of cataract formation. Older age at menarche has been associated with increased risk for cataract and a decreased risk has been shown in women with higher age at menopause [8,9]. Previous studies demonstrate similar risk of cataract for premenopausal women and men at the same age, whereas postmenopausal women exhibit higher risk of cataract than men [6,10-12]. It has therefore been suggested that the increased risk of cataract for women is due to the reduction, rather than the absolute concentration, in estrogen levels after menopause. In Table 1, the concentration of the major endogenous estrogen, 17β-estradiol, is shown for pre- and postmenopausal women and for men. As for the influence of exogenous estrogen on cataractogenesis, data are inconsistent whether or not the use of hormone replacement therapy (HRT) is associated with increased risk of cataract. In some of the studies where protection of HRT against cataract was found [8,13,14], this effect could not be confirmed in follow-up studies [15-17]. In a population based case-control study, the use of estrogen-only preparations have shown protective effects on cataract development [18]. Estrogen therapy has also shown protective effects on nuclear cataract [19] and another study shows similar results for longer duration of estrogen treatment [20]. Although several studies indicate a decreased risk of cataract from HRT, there are also studies showing the opposite [21]. Conflicting data also exist regarding the premenopausal use of estrogens (oral contraceptives) and risk of cataract [13,16,22]. Further support for the impact of hormones on cataractogenesis comes from studies demonstrating increased risk of cataract for women treated with anti-estrogens such as tamoxifen [23,24]. In addition, androgen deprivation in the treatment of prostate cancer has been linked to increased risk of cataract, showing that hormonal status may be important in cataractogenesis in both genders [25].

**Table 1:** Reference range for 17β-estradiol in men and women.

**Women (menstrual cycle phases)**	**17β-estradiol pg/ml (pmol/l)**
Follicular	21–251 (77–921)
Periovulatory	38–650 (139–2390)
Luteal	21–313 (77–1150)
Postmenopausal	<28 (<104)
**Men**	11–44 (40–162)

The serum concentration of the major endogenous estrogen, 17β-estradiol, is shown for pre- and postmenopausal women and for men. Reference range from Sahlgrenska University Hospital, Gothenburg, Sweden.

The mechanism for estrogen-mediated protection against cataract formation is not fully elucidated, although it has been suggested that it is mainly due to anti-oxidative properties of estrogen. It is widely recognized that oxidative stress is a major cause of cataract and estrogen exhibits protective effects against oxidative stress in cultured lens epithelial cells, where it was shown to preserve mitochondrial function, ATP levels and cell viability [26]. However, estrogen has also been shown to confer protection against TGF-β-induced cataract in organ-cultured lenses and to protect against radiation-induced lens opacities in an animal model of cataract [27,28].

In addition to antioxidative effects, it is well known that estrogen promotes proliferation in some cell types and contexts. The most common postoperative complication after cataract surgery, posterior capsular opacification (PCO), is caused by proliferation of residual lens epithelial cells on the lens capsule. The effects of estrogen on proliferation is thus important, especially since female gender has been associated with increased risk of PCO [29].

The purpose of this study was to examine the effects of 17β-estradiol on proliferation, cell death and oxidative stress in cultured human lens epithelial cells (HLECs).

## Methods

Human lens capsule epithelium specimens, usually 5 mm in diameter, were obtained during cataract surgery at the Eye Clinic, Sahlgrenska University Hospital, Mölndal, Sweden, after obtaining informed consent. The lens capsule epithelium specimens were put directly into medium, RPMI-1640 with 10% fetal bovine serum supplemented with 100 U/ml penicillin, 0.1 mg/ml streptomycin and 2 mM L-glutamine, and stored at room temperature for a maximum of 4 days. The specimens were then transferred to 24-well culture dishes (TPP, Trasadingen, Switzerland) at 37 °C in a humidified 5% CO_2_ incubator for 1 week to allow attachment of the capsules. After HLECs had migrated about 5 mm from the capsular edge the cells were subcultured by 0.25% trypsin/EDTA treatment and subsequently stored at −80 °C. The study was approved by The Regional Ethics Committee in Gothenburg, Västra Götaland County, Sweden, and the tenets of the Declaration of Helsinki were followed. All chemicals used were acquired from Sigma-Aldrich Corporation (St.Louis, MO), if nothing else is indicated. In all experiments, three or more cell lines derived from native HLECs were used. Each cell line was subcultured from one individual and passages IV–XV were used for experiments. Morphology of the HLECs exhibited a normal epithelial pattern, indicating that although the cells were derived from cataractous lenses, no gross deviations from normal lens epithelial cell appearance were present. HLECs from both female and male patients were used but no significant difference in results was seen between cells from different genders.

For morphological studies, cells were seeded on chamber slides (Lab-Tek^TM^ Nunc, Rochester, NY). For biochemical measurements, HLECs were cultured in medium RPMI-1640 in a white 96-well plate with transparent bottom (Costar Corp., Cambridge, MA) until a confluent monolayer was obtained. Absorption (optical density) was measured on a microplate reader (E-max) using SOFTmax version 3.1 as software (Molecular Devices, Sunnyvale, CA). Fluorescence-based assays were measured on a microplate reader (SPECTRAmax GEMINI) using SOFTmax PRO version 4.8 as software (Molecular Devices). Prior to biochemical assays or morphological studies, cells were washed with phosphate-buffered saline (PBS) without calcium and magnesium, after which the medium was changed to serum-free RPMI-1640 without phenol red, supplemented with 100 U/ml penicillin, 0.1 mg/ml streptomycin and 2 mM L-glutamine. Stock solution of 17β-estradiol (10 mM) was prepared in 99.5% ethanol. HLECs were incubated in triplicates with 17β-estradiol (0.0001, 0.001, 0.01, 0.1, 1 and 10 µM) in serum-free RPMI-1640 for 24 h at 37 °C in a humidified 5% CO_2_ incubator. For experiments where the antioxidative effect of 17β-estradiol was studied, HLECs were preincubated with 17β-estradiol for 4 h before addition of H_2_O_2_ for 24 h.

### Cell viability and proliferation

MTT (3-[4, 5- dimethylthiazolyl-2]-2, 5-diphenyltetrazolium bromide) is cleaved by mitochondrial dehydrogenases to formazan crystals in metabolically active cells and this method was used to detect viable cells. MTT diluted 1:10 from a stock solution of 5 mg/ml, was added to the 96-well plate and the cells were then incubated for 4 h, after which formazan crystals were solubilized in DMSO. Absorption was measured and the difference between the sample wavelength (570 nm) and the reference wavelength (650 nm) was calculated and the proportion of viable cells was expressed as percentage of control.

Morphology of cell nuclei in HLECs was studied to detect mitosis and apoptosis. Cultured HLECs were fixed in 4% paraformaldehyde in PBS (HistoLab, Gothenburg, Sweden) for 30 min, after which cells were stained with Hoechst 33342 (Hoechst, Frankfurt, Germany) at a final concentration of 5 μg/ml for 15 min at 37 °C. The coverslips were then mounted on chamber slides with Dako’s fluorescent mounting medium (Dako Co., Glostrup, Denmark) followed by counting of stained cells in a fluorescence microscope (Nikon Eclipse TE300; Nikon, Tokyo, Japan). At least 300 cells in three different fields were counted and the number of mitotic/apoptotic nuclei in relation to the total number of cells was determined and expressed as percentage of control.

### Cell death and apoptosis

Hoechst 33342 was used to morphologically detect apoptotic nuclei, following the same protocol as above. In addition, apoptosis was detected and quantified using a caspase-3 assay. After the incubation period with 17β-estradiol, the cells were centrifuged at 350× g for 5 min. The medium was removed, and the well plates were immediately frozen at −152 °C for at least 30 min. The frozen HLECs were thawed, and 100 μl of 0.2% CHAPS-containing buffer including the protease inhibitors, trypsin inhibitors from chicken egg white (final concentration 5 μg/ml), pepstatin (0.5 μg/ml), leupeptin (1.25 μg/ml), and PMSF (0.5 mM) were added. The cells were incubated with the inhibitor-containing CHAPS buffer for 30 min at room temperature after which 20 μl was removed for protein determination.

The synthetic fluorogenic substrate, Ac-Asp-Glu-Val- Asp-AMC (Ac-DEVD-AMC; from Bachem, Bubendorf, Switzerland), used for caspase-3 determination was diluted from a 10 mM stock solution in water to 50 μM in Tris-HCl (pH 7.3), 100 mM NaCl, 5 mM EDTA, 1 mM EGTA, and 3 mM NaN_3_, yielding a final concentration of 25 μM Ac-DEVD-AMC in the assay. Dithiothreitol was added to a final concentration of 2 mM. At the start of the proteolytic assay, 100 μl of substrate was added to the 96 well plate containing 100 μl of cell lysate in CHAPS buffer. The fluorescent cleavage product of the substrate was measured during 2 h (Ex 380 nm, Em 460 nm) and V_max_ was determined in the linear interval. Proteolytic activity is expressed as the increase in relative fluorescence units per second and gram of protein (RFU/s/g). Aliquots of 20 μl HLECs lysate with 0.2% CHAPS buffer were taken for protein determination using the BCA protein assay reagent (Pierce Perbio Science UK Limited, Cheshire, UK) with BSA as the standard and absorption was measured at 570 nm.

Propidium iodide (PI) was used to determine the percentage of non-viable cells. PI was diluted 1:50 from a stock solution of 1 mg/ml in RPMI-1640 without phenol red, and added to the cells for 3 min, after which fluorescence was measured (Ex 540 nm, Em 620 nm). To determine the total number of cells 0.2% CHAPS-containing buffer was added and the cells were frozen at −80 °C. After lysis of cells, HLECs were thawed and relative cell density was measured. This method was also used as a reference for relative cell density in other methods in this study i.e., reactive oxygen species (ROS) and glutathione levels.

### Intracellular ROS levels, glutathione and mitochondrial membrane potential

The change in peroxide levels in cells exposed to 17β-estradiol was measured using the non-fluorescent, dichlorofluorescin diacetate (DCFH-DA; 20 μM). In the cell, DCFH-DA is cleaved by esterases yielding polarized DCFH, which then is oxidized to fluorescent DCF by several ROS including different peroxides (but not O_2_^•-^or H_2_O_2_), therefore we refer to the amount formed DCF as a measurement of the peroxide levels in the cell. The cells were incubated with DCFH-DA for 30 min at 37 °C and peroxide levels was measured (Ex 490 nm, Em 535 nm). Changes in peroxide levels are expressed as RFU/cell density, using PI-labeling of cells to compensate for differences in cell density.

Superoxide levels in HLECs was measured using dihydroethidium (HET), which is oxidized by superoxides to the fluorescent substance ethidium, and changes in ethidium concentration can hence be used as a measure of superoxide levels. After exposure to 17β-estradiol and after subsequent rinsing in PBS, the cells were preloaded with 5 μM HET for 10 min at 37 °C, then HET was removed, the cells rinsed in PBS and RPMI-1640 without phenol red was added. Changes in superoxide levels were measured after incubation in 37 °C for 30 min (Ex 510 nm, Em 600 nm). Changes in superoxide levels are expressed as RFU/cell density.

The level of reduced glutathione (GSH) levels in HLECs was determined using monochlorobimane (MCB; 25μM), which forms a fluorescent conjugate together with GSH, and was measured after 2 h (Ex 380 nm, Em 460 nm). Changes of GSH levels are expressed as RFU/cell density.

An additional method to examine redox status of the HLECs in this study was to determine changes in mitochondrial membrane potential using 1 µM JC-1 (5,5′,6,6’-tetrachloro-1,1’,3,3′- tetraethylbenzimidazolylcarbocyanine iodide). HLECs were incubated with JC-1 for 15 min at 37 °C, after which the dye was removed, the cells were rinsed in PBS and serum-free RPMI-1640 was added. Oxidative stress usually causes a disruption of the mitochondrial membrane potential and the JC-1 dye indicates mitochondria depolarization by a decrease in the red to green fluorescence intensity ratio. The green (Ex 485 nm, Em 535 nm) and red (Ex 540 nm, 590 nm) JC-1 signals were measured. Valinomycin was used as a positive control in all experiments (not shown).

### Statistics

All data are from triplicate samples and are shown as mean±SEM. Each experiment was run at least three times to confirm reproducibility. Statistical analysis was performed using ANOVA with Dunnett’s as post-hoc. A p-value of less than 0.05 was considered statistically significant. SPSS, version 18.0 (SPSS Inc., Chicago, IL) for Mac OS X, was used as statistical software.

## Results

### Effects of 17β-estradiol in cultured HLECs

**Figure 1:**
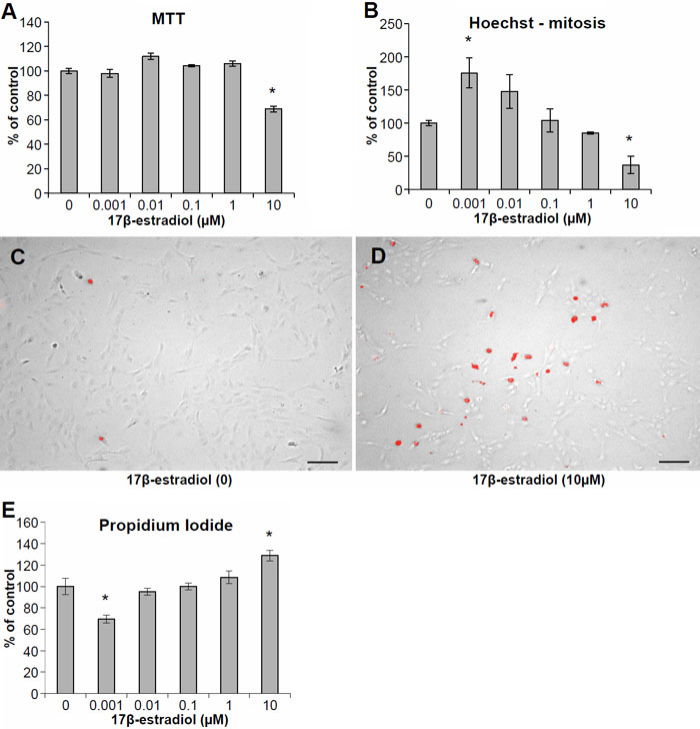
Effects on proliferation and cell death in human lens epithelial cells (HLECs) exposed to 17β-estradiol at different concentrations for 24 h. **A**: The change in number of viable cells (% of control) was determined by the MTT colorimetric assay. **B**: Difference in the number of mitotic cells (% of control) as evident after staining with Hoechst 33342. **C**, **D**: HLECs stained with PI after exposure to 10 µM 17β-estradiol and corresponding control. **E**: Differences in the number of dead cells (% of control) determined by labeling with PI. Mean±SEM is shown. Experiments were performed three times in triplicates (n=3) and one representative experimental run for each method is shown. *p<0.05 as compared to control without 17β-estradiol (0) exposure. Scale bar=100 µm.

**Figure 2:**
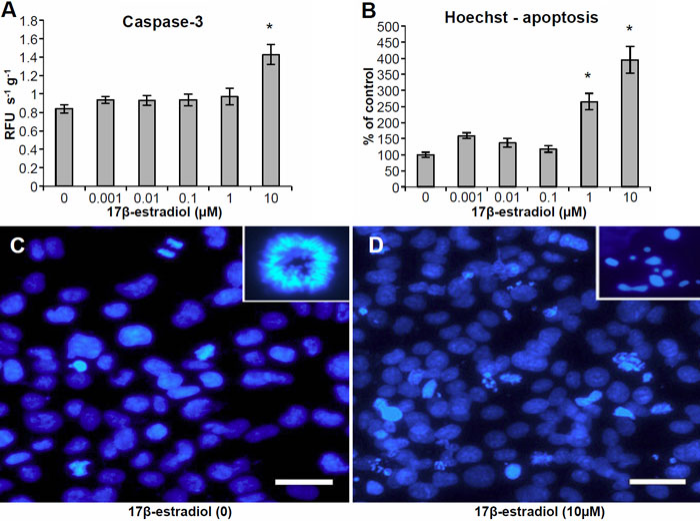
Apoptosis in human lens epithelial cells (HLECs) exposed to 17β-estradiol at different concentrations for 24 h. **A**: Increase in Caspase-3 activity after 17β-estradiol exposure. **B**: Increased number of apoptotic cells (% of control) as evident by staining with Hoechst 33342. Mean±SEM is shown. Experiments were performed three times in triplicates (n=3) and one representative experimental run for each method is shown. *p<0.05 as compared to control without 17β-estradiol (0) exposure. **C**, **D**: HLECs stained with Hoechst after exposure to 10 µM 17β-estradiol and corresponding control. Insets show mitotic and apoptotic nucleus respectively. Scale bar=50 µm.

We performed dose–response experiments with 17β-estradiol at concentrations between 0.1 nM and 10 µM. At 10 µM 17β-estradiol, a significantly lower number of viable cells was observed using the MTT-assay (Figure 1A) as well as decreased levels of mitotic nuclei as evident by Hoechst-staining (Figure 1B). At lower concentrations of 17β-estradiol (1 nM), an increase in the number of mitotic nuclei (Figure 1B) and decrease in cell death (Figure 1E) was observed with Hoechst and PI, respectively. An increase in the relative number of non-viable cells was measured with PI at 10 µM 17β-estradiol (Figure 1C-E). Caspase-3 activity was increased at 10µM 17β-estradiol (Figure 2A) and at 1–10 µM, there was also an increase in the number of apoptotic nuclei as evident by staining with Hoechst 33342 (Figure 2B-D).

**Figure 3:**
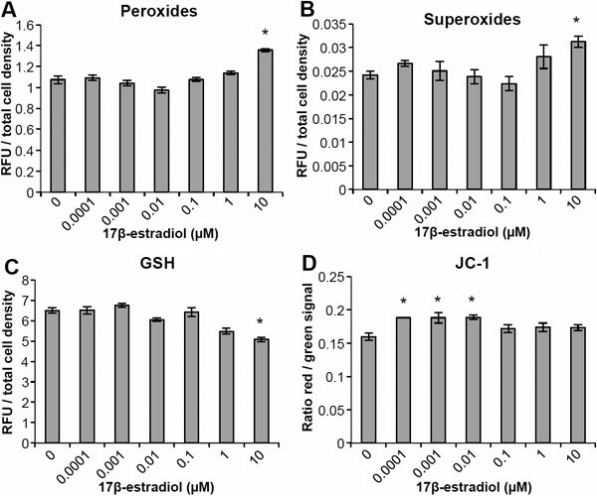
Effects on reactive oxygen species levels and mitochondrial membrane potential in human lens epithelial cells (HLECs), exposed to 17β-estradiol at different concentrations for 24 h. **A**: An increase in peroxide levels was observed at the highest concentration used, 10 µM. **B**: Elevated levels of superoxides was evident at 10 µM 17β-estradiol. **C**: The levels of reduced glutathione (GSH) decreased at 10 µM 17β-estradiol. **D**: An increase in mitochondrial membrane potential (JC-1) at lower concentrations, 0.1–10 nM, of 17β-estradiol. Changes are expressed as the ratio of red signal and green signal. Mean±SEM is shown. Experiments were performed three times in triplicates (n=3) and one representative experimental run for each method is shown. *p<0.05 as compared to control without 17β-estradiol (0) exposure.

At the highest concentration of 17β-estradiol used in these experiments (10 µM) both an increase in peroxide levels (Figure 3A) and in superoxide levels (Figure 3B) was observed. There was also a decrease in GSH levels (Figure 3C) at the same concentration. An increase in the mitochondrial membrane potential (Figure 3D) was seen in the lower range of 17β-estradiol concentrations used, 0.1–10 nM.

### Oxidatively stressed HLECs exposed to 17β-estradiol exhibit reduced ROS levels

**Figure 4:**
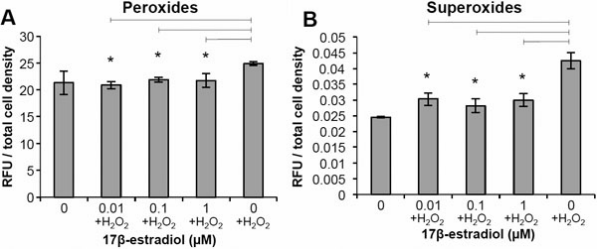
Antioxidative effects of 17β-estradiol against H_2_O_2_ exposure. Human lens epithelial cells (HLECs) were preincubated with 17β-estradiol for 4 h and then simultaneously exposed to 25 µM H_2_O_2_ and 17β-estradiol for 24 h. **A**: A decrease in peroxide levels at low concentrations of 17β-estradiol (0.01–1 µM) was demonstrated. **B**: Superoxide levels were decreased at 0.01–1 µM 17β-estradiol. Mean±SEM is shown. Experiments were performed three times in triplicates (n=3) and one representative experimental run for each method is shown. *p<0.05 as compared to control without 17β-estradiol (0) exposure.

We performed dose–response experiments with H_2_O_2_ to oxidatively stress HLECs and to determine the lowest concentration that caused oxidative stress to the cells by monitoring peroxide, superoxide and glutathione levels (data not shown). The concentration chosen, 25 µM H_2_O_2_, was then used for subsequent experiments. To examine the effect of 17β-estradiol in oxidatively stressed HLECs, the cells were preincubated with 17β-estradiol for 4 h and then simultaneously exposed to 25 µM H_2_O_2_ and different concentrations of 17β-estradiol for 24 h. At low concentrations, 0.01 to 1 µM, 17β-estradiol protected against oxidative stress in HLECs exposed to 25 µM H_2_O_2_, as evident by decreased peroxide and superoxide levels as compared to control cells exposed to H_2_O_2_ alone (Figure 4A,B). The peroxide levels in HLECs incubated with 17β-estradiol were reduced to the same level as in cells not exposed to H_2_O_2_. Superoxide levels were also decreased but not to the same extent as in cells without H_2_O_2_ exposure. However, 17β-estradiol did not reverse the effects of 25 µM H_2_O_2_ on mitochondrial membrane potential (JC-1) or GSH levels (data not shown).

## Discussion

In the present study several techniques were used to determine proliferation and cell viability. The caspase-3 activity assay used in this study is highly specific for apoptosis and together with the proportion of apoptotic cell nuclei, as determined with Hoechst staining, a reliable determination of the apoptotic response to 17β-estradiol could be made. Propidium iodide stains both apoptotic and necrotic cells, whereas the MTT assay is a measure of the total number of viable cells and is thus affected both by changes in proliferation and in cell death, including apoptosis and necrosis. The three methods above were hence chosen to complement each other.

The present data demonstrates increased mitotic activity in HLECs exposed to physiologic concentrations of 17β-estradiol. Higher concentrations of 17β-estradiol caused cell death by apoptosis, as evident by increased number of apoptotic cell nuclei and caspase-3 activation. Although estrogen is mainly associated with a proliferative response, required for growth of hormone-sensitive cancer, the opposite has also been demonstrated, hence the expression “the estrogen paradox” [30]. In contrast to previous studies, decreased risk of breast cancer was demonstrated in a randomized controlled trial of postmenopausal women using HRT [31] and laboratory work has demonstrated estradiol-induced apoptosis in hormone-dependent breast cancer cells, but only after a long period of estrogen-deprivation [32]. Thus, clinical and experimental studies show estrogens to be key regulators in tissue homeostasis by sensitizing cells to both mitogenic and apoptotic signals and by inducing expression of growth factors and cytokines [33]. The exact mechanism for estrogen-mediated apoptosis is not clear; both extrinsic and intrinsic apoptotic pathways exist, the latter being triggered by mitochondrial collapse of membrane potential. Our data demonstrate caspase-dependent apoptosis at pharmacological concentrations of 17β-estradiol, without mitochondrial depolarisation.

The stimulatory effect of physiologic concentrations of estrogen on proliferation of cultured HLECs in this study is especially interesting from an ophthalmologic view since female gender has been suggested as a risk factor for developing PCO [29]. Recent data from our group show that HLECs in primary cultures of capsule-epithelium specimens exhibit slightly higher rate of cell growth if derived from female cataract patients than from male [34].

Oxidative stress is well recognized as a cataractogenic factor. Increased concentration of H_2_O_2_ has been demonstrated in aqueous humor from cataract patients [35]. The H_2_O_2_-concentration, 25 µM, used in the present experiments was well within the reported range; <10 to >660 µM [35,36] and was chosen since this level of H_2_O_2_ induced ROS-production but did not cause apoptosis (data not shown). Moreover, all experiments with H_2_O_2_-exposure to cultured HLECs were performed in serum-free medium, since studies by others have shown substantial reduction of H_2_O_2_ by 20% serum alone [37].

Exposure of cultured HLECs to pharmacological concentrations of 17β-estradiol (10 µM) in this study resulted in increased levels of peroxides and superoxides, as well as decreased GSH levels. Mitochondrial membrane potential was not affected by 10 µM 17β-estradiol, indicating that this increased ROS production did not hamper mitochondrial function. Instead, physiologic concentrations of 17β-estradiol (0.1–10 nM) further stabilized the mitochondrial membrane potential as evident by JC-1-staining. Similar or slightly higher concentrations of 17β-estradiol (0.01–1 µM) also protected against H_2_O_2_-induced oxidative stress, as shown by decreased levels of peroxides and superoxides. This effect was more pronounced for peroxides where levels were comparable with non-oxidatively stressed cells, whereas the reduction of superoxides was significant but not as prominent as for peroxides. Estrogen has been ascribed both pro-and anti-oxidative properties; for a recent review see Kumar et al. [38]. By stimulating transcription of mitochondrial structural proteins, respiratory chain metabolism is increased and hence also the production of ROS. However, the phenolic hydroxyl group of 17β-estradiol can act as a ROS scavenger, thus lowering the levels of peroxides and superoxides. Moreover, 17β-estradiol has been shown to stabilize mitochondrial membrane potential, preventing the release of pro-apoptotic signals such as cytochrome C and Apaf-1 [39]. Wang et al. [26] reported estrogen-mediated protection against H_2_O_2_-induced oxidative stress in a human lens epithelial cell line (HLE-B3 cells). Pretreatment with 17β-estradiol caused a dose-dependent preservation of mitochondrial potential, intracellular ATP-levels and increased cell viability. However, no effects on ROS levels were seen in the same study at concentrations ranging from 1 nM to 10 µM 17β-estradiol, when using 100 µM H_2_O_2_ to oxidatively stress lens epithelial cells. This is thus in contrast to our data where 17β-estradiol reversed the increase in peroxide and superoxide levels seen in HLECs exposed to 25 µM H_2_O_2_. In addition, the present study did not detect estrogen-mediated protection of mitochondrial membrane potential against H_2_O_2_–induced oxidative stress.

Additional proposed anti-oxidative mechanisms for estrogens include upregulation of anti-oxidative enzymes, such as manganese superoxide dismutase (SOD-2) and glutathione peroxidase, the latter resulting in higher GSH-levels in mitochondria from female as opposed to male rats [40]. In our study, no restoration of GSH concentration could be observed in oxidatively stressed HLECs after pretreatment with 17β-estradiol. As for SOD-2, Gottipati et al. [41] showed a rapid and transient increase in activity in HLE-B3 cells 90 min after exposure to 17β-estradiol, without an accompanying increase in SOD-2 mRNA or protein levels, suggesting a non-genomic action of estradiol. However, similar experiments in other cell types, such as vascular smooth muscle cells, demonstrated estrogen-induced expression of both SOD-2 and extracellular superoxide dismutase (SOD-3) [42].

The present study demonstrates anti-oxidative effects at low (physiologic) levels and pro-oxidative effects at high (pharmacological) concentrations of 17β-estradiol. Studies on the neuroprotective effects of estrogen, i.e., beneficial effects against Parkinson disease and Alzheimer disease, suggest that estrogen-mediated neuroprotection is estrogen receptor (ER)-dependent at physiologic levels and ER-independent at pharmacological concentrations of the hormone [43]. There are two distinct types of estrogen receptors, ERα and ERβ, both of which have been demonstrated in lens epithelial cells [44]. Most studies performed so far on ERs in lens epithelial cells have used transformed cell lines. However, the relative abundance and preferential subcellular distribution of the two ERs and their respective isoforms exhibit substantial variations between species as well as between freshly isolated capsulorhexis specimens and cultured lens epithelial cells and also in native versus transformed cells [45,46]. The intracellular localization and ratio between ERα and ERβ have been suggested to account for much of the differences seen in estrogen response in various cell types and may also explain discrepancies between the present study, performed on native human lens epithelial cells solely, and other studies on this subject.
